# MOFs—Combining Fully Synthetic Injectable Hydrogel Scaffolds Exhibiting Higher Skeletal Muscle Regenerative Efficiency than Matrigel

**DOI:** 10.3390/gels11070514

**Published:** 2025-07-02

**Authors:** Sobuj Shahidul Islam, Tatsuya Dode, Soma Kawashima, Myu Fukuoka, Takaaki Tsuruoka, Koji Nagahama

**Affiliations:** Department of Nanobiochemistry, Frontiers of Innovative Research on Science and Technology (FIRST), Konan University, 7-1-20 Minatojima-Minamimachi, Chuo-ku, Kobe 650-0047, Japan; d2161501@s.konan-u.ac.jp (S.S.I.); tatsuya.dd@icloud.com (T.D.); m2461007@s.konan-u.ac.jp (S.K.); h8.kinki.meishinkai@gmail.com (M.F.)

**Keywords:** injectable hydrogels, MOFs, scaffolds, tissue engineering, skeletal muscle tissue

## Abstract

Due to its sarcoma-derived origin and the associated carcinogenic risks, as well as its lack of tissue-specific extracellular matrix biochemical cues, the use of the injectable gel scaffold Matrigel is generally restricted to research applications. Therefore, the development of new fully synthetic injectable gel scaffolds that exhibit performance comparable to Matrigel is a high priority. In this study, we developed a novel fully synthetic injectable gel scaffold by combining a biodegradable PLGA-PEG-PLGA copolymer, clay nanoparticle LAPONITE®, and L-arginine-loaded metal–organic frameworks (NU-1000) at the nano level. An aqueous solution of the developed hybrid scaffold (PLGA-PEG-PLGA/LAPONITE®/L-Arg@NU-1000) exhibited rapid sol–gel transition at body temperature following simple injection and formed a continuous bulk-sized gel, demonstrating good injectability. Long-term sustained slow release of L-arginine from the resultant gels can be achieved because NU-1000 is a suitable reservoir for L-arginine. PLGA-PEG-PLGA/LAPONITE®/L-Arg@NU-1000 hybrid gels exhibited good compatibility with and promoted the growth of human skeletal muscle satellite cells. Importantly, in vivo experiments using skeletal muscle injury model mice demonstrated that the tissue regeneration efficiency of PLGA-PEG-PLGA/LAPONITE®/L-Arg@NU-1000 gels is higher than that of Matrigel. Specifically, we judged the higher tissue regeneration efficacy of our gels by histological analysis, including MYH3 immunofluorescent staining, H&E staining, and Masson’s trichrome staining. Taken together, these data suggest that novel hybrid hydrogels could serve as injectable hydrogel scaffolds for in vivo tissue engineering and ultimately replace Matrigel.

## 1. Introduction

Metal–organic frameworks (MOFs) are hybrid materials composed of metal ions and organic ligands that form porous structures. MOFs offer distinct advantages over other frameworks, including exceptional compositional and structural diversity, high porosity, and ultrahigh specific surface area [1,2,3,4]. Given their unique structural potential, MOFs are employed in a broad range of applications, including gas storage [5,6], catalysis [7,8], and sensing [9,10]. Over the past decade, interest in the use of MOFs for biomedical applications has grown. The structural potential represented by the high surface area and exceptional porosity of MOFs makes them particularly well-suited for use as carriers in drug delivery systems, as they offer the potential for sustained, controlled release of small-molecule drugs [11,12,13].

The application of MOFs in tissue engineering has gained significant recent attention due to their unique biocompatibility characteristics [14,15,16,17,18]. Several studies have explored combining MOFs with bulk materials in order to generate three-dimensional scaffolding materials. Mechanically, such combinations provide an optimal extracellular microenvironment that supports cell survival, proliferation, and organization, enabling these materials to play a crucial role in tissue engineering [19,20,21,22,23]. Hydrogels are particularly suitable for use as three-dimensional scaffolds in tissue engineering due to their good biocompatibility, high water content, and soft, flexible nature, which mimics the extracellular matrix of natural tissues. A number of other studies have also reported the development of MOF-containing combined hydrogels [24,25,26,27,28]. For instance, using a simple solvothermal reaction, Wang et al. developed novel donut-like MOFs composed of copper–nicotinic acid (CuNA). The rough surface of CuNA particles enhanced the loading and release of basic fibroblast growth factor (bFGF). Combining CuNA with GelMA produced a light-responsive composite hydrogel that exhibited excellent mechanical properties and superior biocompatibility and bioactivity, effectively promoting wound closure. By 14 days, bFGF-loaded CuNA@GelMA gels exhibited 98% epithelium coverage, the formation of new blood vessels, and mild inflammatory responsive wound closure [29,30]. The neobavaisoflavone and polydopamine–hyaluronic acid (NBIF@ZIF-8/PHG) hydrogels developed by Jiang et al. promoted the repair of cartilage defects. The ICRS gross observation scores of NBIF@ZIF-8/PHG gels were significantly higher than those of control PHG groups. Cartilage regenerated through the use of NBIF@ZIF-8/PHG exhibited a visible transverse mark between the cartilage and subchondral bone [31].

Compared with the hydrogels described above, injectable gels have emerged as highly promising scaffold materials in tissue engineering. Extensive research conducted over the past few decades has led to the development of a wide range of hybrid injectable gels. This approach facilitates the formation of a self-regenerative environment in situ and represents a minimally invasive way to promote the healing of surrounding injured areas [32,33,34]. Matrigel is a basement-membrane matrix extracted from Engelbreth–Holm–Swarm mouse sarcoma cells, and it has been used for more than four decades in a wide range of cell culture applications. As Matrigel also exhibits a temperature-responsive sol–gel transition, it can be used as an injectable gel. Matrigel is considered an excellent scaffolding material for cell transplantation applications and strongly promotes tissue regeneration. However, the applications of Matrigel are limited to cell biology research, therapeutic cell manufacturing, and drug discovery due to its complex, poorly defined, and variable composition. Reported variations in the mechanical and biochemical properties within single batches of Matrigel and between batches have led to uncertainty in cell culture experiments and a lack of reproducibility. Moreover, Matrigel does not respond well to physical or biochemical manipulations, making it difficult to fine-tune the matrix in order to promote intended cell behaviors and achieve specific biological outcomes [35,36,37,38,39]. One paper suggested that because Matrigel is unlikely to receive FDA approval, it is not suitable for use in human tissue engineering [37]. Therefore, many researchers have attempted to develop synthetic hydrogel scaffolds that exhibit tissue regeneration properties comparable to those of Matrigel. Although a variety of synthetic polymers and nanomaterials have been used to develop these scaffolds, success has been limited because of the presence of significant challenges. Nanomaterials such as graphene or MOFs may also induce oxidative stress or immune responses due to their surface reactivity and size. Moreover, controlled degradation is challenging to achieve, potentially leading to premature material breakdown or chronic toxicity. Additionally, the scale-up and reproducibility of nanomaterials remain problematic because of batch variability and the complexity of their synthesis [40,41].

We developed an injectable gel that is generated via the hierarchical self-assembly of disk-shaped clay nanoparticles (LAPONITE^®^) and biodegradable poly(DL-lactide-*co*-glycolide)-*b*-poly(ethylene glycol)-*b*-poly(DL-lactide-*co*-glycolide) copolymers (PLGA-PEG-PLGA) (Figure S1) [42]. To improve the tissue regenerative capability, we recently modified the material design by incorporating MOFs into the PLGA-PEG-PLGA/LAPONITE^®^ gel [43]. We selected L-arginine (L-Arg) as a bioactive molecule because of its known regenerative role in promoting angiogenesis, which is thought to involve the restoration of impaired nitric oxide (NO) synthesis and associated pathways, with NO bioavailability ultimately enhanced via the activity of endothelial nitric oxide synthase [44,45]. Moreover, NU-1000 was selected as the MOF because of its high biocompatibility and high stability in water at body temperature, as well as its porous size match with the size of L-Arginine [46,47,48,49]. Thus, large amounts of L-Arg can be loaded into the interior pores of NU-1000, facilitating the generation of L-Arg–encapsulating, NU-1000–combined PLGA-PEG-PLGA/LAPONITE^®^ gels (PLGA-PEG-PLGA/LAPONITE^®^/L-Arg@NU-1000). Sustained slow release of L-Arg from the combined hydrogels was achieved because of the efficient carrier functionality of NU-1000, enabling the highly efficacious promotion of angiogenesis.

**Figure 1:**
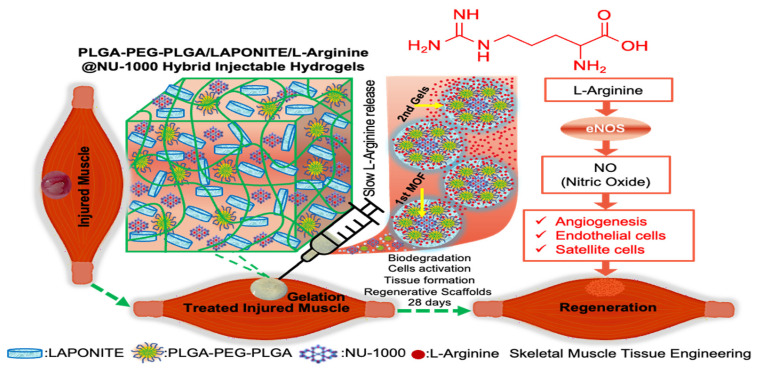
Schematic illustration of the structure and function of the developed PLGA-PEG-PLGA/LAPONITE^®^/L-Arg@NU-1000 injectable hydrogels for the promotion of skeletal muscle tissue regeneration.

Considering the potential of the PLGA-PEG-PLGA/LAPONITE^®^/L-Arg@NU-1000 gels, we hypothesized that the PLGA-PEG-PLGA/LAPONITE^®^/L-Arg@NU-1000 gels could become a new type of injectable gel scaffold and replace Matrigel for tissue engineering applications (Figure 1), as neovascularization is an essential process in the regeneration and functional recovery of injured tissues. Moreover, L-Arg is known to decrease inflammation and enhance muscle regeneration by modulating various signaling pathways, such as the nuclear factor-κB pathway, leading to accelerated NO production, protein synthesis, and modulation of inflammation [50,51]. Thus, L-Arg could be effective in promoting tissue regeneration using PLGA-PEG-PLGA/LAPONITE^®^ gel (Figure 1).

Many researchers have investigated the muscle repair and regenerative capabilities of injectable gel scaffolds, including Matrigel [52,53,54,55,56,57,58,59,60]. In this context, the present study used skeletal muscle as a model to compare the effect of PLGA-PEG-PLGA/LAPONITE^®^/L-Arg@NU-1000 hydrogels and Matrigel to promote tissue regeneration. The rheological properties, injectability, L-arginine release properties, and in vitro cell compatibility of the PLGA-PEG-PLGA/LAPONITE^®^/L-Arg@NU-1000 hydrogels were evaluated using human skeletal muscle satellite cells (HsKMSCs), and the skeletal muscle regeneration capability was evaluated in vivo. The results of these analyses were compared with those for Matrigel. Notably, the PLGA-PEG-PLGA/LAPONITE^®^/L-Arg@NU-1000 hydrogels demonstrated significantly higher skeletal muscle regeneration capability than Matrigel.

## 2. Results and Discussion

### 2.1. Preparation of PLGA-PEG-PLGA/LAPONITE/L-Arg@NU-1000 Hydrogels

The PLGA-PEG-PLGA copolymer was synthesized via anionic ring-opening polymerization of _L_-Lactide (_L_-LA), _D_-lactide (_D_-LA), and glycolide (GA) in the presence of polyethylene glycol (PEG) as a macroinitiator and tin 2-ethylhexanoate as a catalyst (Figure S2). The molecular composition, molecular weight, and molecular weight distribution were determined by ^1^H-NMR spectroscopy and gel permeation chromatography (GPC) analysis. The specific values for the degree of polymerization (DP) were LA = 8, GA = 4, M_w_ of PEG = 3000 Da, M_n_ of copolymer = 4960 Da, and M_w_ of copolymer = 6300 Da. The molecular weight distribution was M_w_/M_n_ ≤ 1.3%, as summarized in Table S1. PLGA-PEG-PLGA/LAPONITE^®^ gels were prepared by simple mixing of aqueous solutions of PLGA-PEG-PLGA and LAPONITE^®^ at specific concentrations. The PLGA-PEG-PLGA/LAPONITE^®^ gels examined in this study were named according to the respective concentrations of PLGA-PEG-PLGA and LAPONITE^®^. For instance, P3L1.1 refers to a hydrogel consisting of PLGA-PEG-PLGA (3.0%) and LAPONITE^®^ (1.1%). In this study, P3L1.1 hydrogels were incorporated with L-Arg@NU-1000 to fabricate composite gel formulations with varying L-arginine content. Two types of PLGA-PEG-PLGA/LAPONITE^®^/L-Arg@NU-1000 hydrogels were prepared, one containing 0.2 mg of L-arginine and 0.2 mg of NU-1000 per mL of hydrogel, and another containing 0.6 mg of L-arginine and 0.5 mg of NU-1000 per mL of hydrogel, corresponding to a total L-arginine loading of 152 μg/mL in the latter formulation. Additionally, a control hydrogel formulation, P3L1.1/L-Arg (0.2 mg), lacking NU-1000, was prepared. This allowed for comparative analysis with the P3L1.1/L-Arg (0.2 mg) @NU-1000 (0.2 mg) injectable hydrogel.

### 2.2. Rheological Properties of the Hydrogels

**Figure 2:**
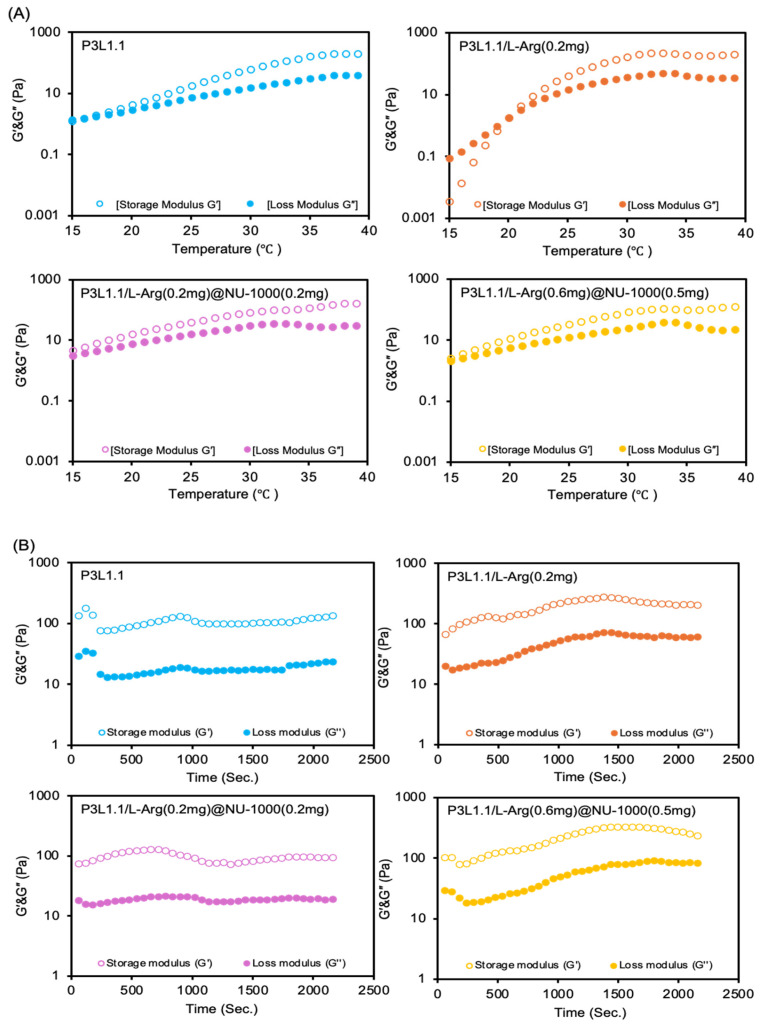
(**A**) Temperature-dependent rheological properties of the PLGA-PEG-PLGA/ LAPONITE^®^/L-Arg@NU-1000 hydrogels and the PLGA-PEG-PLGA/LAPONITE^®^ hydrogel. (**B**) Time-dependent rheological properties of the PLGA-PEG-PLGA/LAPONITE^®^/L-Arg@NU-1000 hydrogels and the control PLGA-PEG-PLGA/LAPONITE^®^ hydrogel measured at 37 °C.

We previously reported the construction of the gel network structure of PLGA-PEG-PLGA/LAPONITE^®^ gels via the hierarchical self-assembly of PLGA-PEG-PLGA nanomicelles and LAPONITE^®^ nanoparticles [61]. Briefly, hydrogen bonds form the main interactions at physical cross-linking points between the hydroxyl groups of LAPONITE^®^ molecules located on the planar surface and the ether oxygen molecules of the PEG segments in the nanomicelles. Notably, although the PLGA-PEG-PLGA nanomicelle solution exhibited reversible thermo-gelation, the PLGA-PEG-PLGA/LAPONITE^®^ composite solution underwent irreversible thermo-gelation due to the resultant physical cross-linking through interactions between LAPONITE^®^ and PLGA-PEG-PLGA nanomicelles. Similarly, NU-1000 harbors surface carboxyl residues associated with the tetra-carboxylate ligand H4TBAPy that form hydrogen bonds with the hydroxyl groups of LAPONITE^®^. Infrared (IR) spectroscopy revealed characteristic absorption bands of the carboxylic group at 1615 cm^−1^ and 1417 cm^−1^. However, when combined with P3L1.1, this characteristic peak completely disappeared. Since the carboxylic group is not expected to interact significantly with the PLGA-PEG-PLGA component, the disappearance of these peaks suggests a possible interaction between the carboxylic groups and Laponite. Given that Laponite possesses various ionic and salt-interacting sites, it is likely that these groups interact with the carboxylic functionalities of NU-1000, leading to the observed spectral changes shown in Figure S3 [62,63,64]. Considering that NU-1000 can interact with LAPONITE^®^, we hypothesized that the gelation behavior of the PLGA-PEG-PLGA/LAPONITE^®^ gels can vary in the presence of different amounts of L-Arg@NU-1000. Therefore, the rheological properties of the PLGA-PEG-PLGA/LAPONITE^®^/L-Arg@NU-1000 gels were analyzed (Figure 2A,B). The gelation temperature was determined by the crossover point of the G′ and G″ values. The gelation temperature and mechanical strength were strongly affected in combination with L-Arg@NU-1000 (Table 1). The gelation temperature decreased in the presence of L-Arg@NU-1000, with values of 19 ± 3.6, 21 ± 1.0, 15 ± 0.6, and 18 ± 3.5 °C for P3L1.1, P3L1.1/L-Arg (0.2 mg), P3L1.1/L-Arg (0.2 mg)@NU-1000 (0.2 mg), and P3L1.1/L-Arg (0.6 mg)@NU-1000 (0.5 mg), respectively. The L-Arg-loading PLGA-PEG-PLGA/LAPONITE^®^ gel, P3L1.1/L-Arg (0.2 mg), exhibited a somewhat higher gelation temperature (20 °C) than that of the P3L1.1 gel (16 °C), indicating that free L-Arg interferes with the gelation reaction of the PLGA-PEG-PLGA/LAPONITE^®^ gel. By contrast, the P3L1.1/L-Arg (0.2 mg)@NU-1000 (0.2 mg) gel showed a lower gelation temperature than the P3L1.1/L-Arg (0.2 mg) gel, even though they had the same amount of L-Arg loaded. This result suggests that L-Arg does not interfere with the gelation reaction after it is loaded into the pores of NU-1000. As mentioned above, the possible interaction between the components of NU-1000 and LAPONITE^®^ may promote the gelation reaction.

The storage moduli (G′) of the hydrogels at 37 °C increased in the presence of L-Arg@NU-1000: the G′ values of P3L1.1, P3L1.1/L-Arg (0.2 mg), P3L1.1/L-Arg (0.2 mg)@NU-1000 (0.2 mg), and P3L1.1/L-Arg (0.6 mg)@NU-1000 (0.5 mg) at 37 °C were 141 ± 41, 181 ± 82, 124 ± 49, and 237 ± 72 Pa, respectively. These results are consistent with the results of the gelation temperature analyses, which showed promotion of the gelation reaction of the PLGA-PEG-PLGA/LAPONITE^®^ gels in the presence of L-Arg@NU-1000. Notably, when the formed gels were kept at 37 °C, a significant decrease in the G′ value of the PLGA-PEG-PLGA/LAPONITE^®^ gels (P3L1.1) was detected, suggesting that dynamic dissociation of the gel network occurs at body temperature. By contrast, no decrease in the G’ value was observed for the P3L1.1/L-Arg (0.2 mg)@NU-1000 (0.2 mg) and P3L1.1/L-Arg (0.6 mg)@NU-1000 (0.5 mg) gels, confirming the stability of the G’ values (Figure 2B). Exceeding 1 mg of NU-1000 incorporation into the hydrogel formulation was found to disrupt gelation behavior, compromising the injectable hydrogels’ structural integrity and performance. Such stability in the gel networks and resultant mechanical strength is considered a preferable property for injectable gels. Native skeletal muscle tissue exhibits a stiffness modulus of approximately 100 kPa. In contrast, our developing injectable hydrogels, P3L1.1/L-Arg (0.6 mg)@NU-1000 (0.5 mg), demonstrated a significantly lower stiffness modulus of 237 Pa. Despite this mechanical mismatch, the hydrogels effectively supported robust skeletal muscle tissue regeneration after 28 days. Furthermore, ensuring mechanical stability of the hydrogel at 37 °C is essential for maintaining structural integrity under physiological conditions. Such stability is particularly advantageous for muscle tissue regeneration, where mechanical signals play a pivotal role in directing cellular responses, enhancing cell adhesion, and supporting the tissue repair process [65].

### 2.3. In Vitro Injectability of the Hydrogels

**Figure 3:**
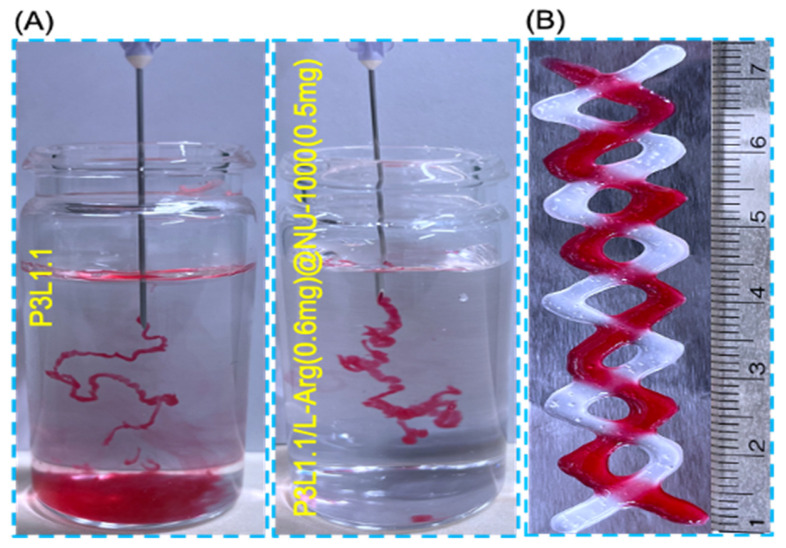
(**A**) Representative images of the injectability of the PLGA-PEG-PLGA/LAPONITE^®^ gel (left) and the PLGA-PEG-PLGA/LAPONITE^®^/L-Arg@NU-1000 gel (right) tested at 37 °C. (**B**) Representative image of the self-standing PLGA-PEG-PLGA/LAPONITE^®^/L-Arg@NU-1000 gel at 37 °C fabricated by simple injection.

For injectable gels, the ability to undergo the sol@gel transition and produce mechanically stable gels even after syringe administration is very important. Therefore, we performed in vitro injectability tests by injecting gel precursor solutions into a large excess of water at 37 °C. Both the P3L1.1 and PLGA-PEG-PLGA/LAPONITE^®^/L-Arg (0.6 mg)@NU-1000 (0.5 mg) total volume of 1 mL hydrogel formulations exhibited comparable profiles to the normal injection profiles. The amounts of L-arginine (0.6 mg) and NU-1000 (0.5 mg) indicate their total amounts contained in 1 mL of the hydrogel formulations. In this experiment, the precursor solutions were stained with red dye to improve visibility (Figure 3A). The injectability of the P3L1.1/L-Arg (0.6 mg)@NU-1000 (0.5 mg) gels was specifically compared with that of the non-combined P3L1.1 gels. For the P3L1.1 gels, some of the P3L1.1 precursor solutions formed small, fragmented gels and dissolved in water, indicating that the P3L1.1 precursor solutions were unable to support a rapid temperature-responsive gelation reaction. The gelation temperatures of the P3L1.1 hydrogel (19 °C) and the P3L1.1/L-Arg (0.6 mg)@NU-1000 (0.5 mg) formulation (21 °C) showed only a slight variation (2 °C), indicating that such a difference does not significantly affect the injectability of the hydrogel system. A total volume of 1 mL of the P3L1.1 hydrogel was fragmented into gel segments of approximately 300 μL each. By contrast, the P3L1.1/L-Arg (0.6 mg)@NU-1000 (0.5 mg) solutions formed continuous bulk gels without fragmentation, demonstrating that combining L-Arg@NU-1000 and the P3L1.1 gels provides improved injectability. This may be a positive effect due to the interaction between NU-1000 and LAPONITE^®^. Notably, the P3L1.1/L-Arg (0.6 mg)@NU-1000 (0.5 mg) solutions formed self-standing gels that maintained their structural integrity when 2D printed onto the surface of a hot plate held at 37 °C (Figure 3B). These results indicate that the P3L1.1/L-Arg (0.6 mg)@NU-1000 (0.5 mg) gels are suitable not only for use as injectable gels for transplants but also as an ink for 3D bioprinting.

### 2.4. L-Arginine Release Profiles of the Hydrogels

**Figure 4:**
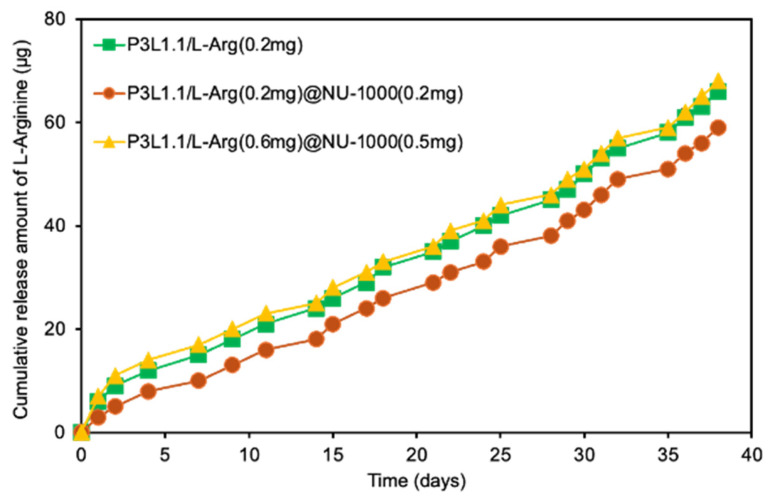
Release profiles of L-arginine from the PLGA-PEG-PLGA/LAPONITE^®^/L-Arg@NU-1000 gels with different amounts of L-arginine loading and the control PLGA-PEG-PLGA/LAPONITE^®^ gel.

Next, we investigated how the combination with NU-1000 affects the release of L-Arg from the gels. This experiment compared the release kinetics of L-Arg from the original P3L1.1 gels and the P3L1.1/L-Arg@NU-1000 gels. As shown in Figure 4, all the gels tested showed sustained release of L-Arg without an initial burst release. Notably, although the same amount of L-Arg was loaded into the P3L1.1 gels [P3L1.1/L-Arg (0.2 mg)] and the P3L1.1/L-Arg (0.2 mg)@NU-1000 (0.2 mg) gels, the rate of L-Arg release from the P3L1.1/L-Arg (0.2 mg) gels was clearly higher than the rate of release from the P3L1.1/L-Arg (0.2 mg)@NU-1000 (0.2 mg) gels. Even when the amount of L-Arg loaded and the amount of NU-1000 combined with the gels were increased, the P3L1.1/L-Arg (0.6 mg)@NU-1000 (0.5 mg) gels exhibited sustained release of L-Arg. Even when the amount of L-Arg loaded and amount of NU-1000 combined with the gels were increased, the P3L1.1/L-Arg (0.2 mg), P3L1.1/L-Arg (0.2 mg)@NU-1000 (0.2 mg) and P3L1.1/L-Arg (0.6 mg)@NU-1000 (0.5 mg) gels exhibited sustained release (49 μg) of L-Arg above (65%, 55%, and 75%) at 38 days. These results indicate that NU-1000 acts as an intragel reservoir for L-Arg. Notably, the sustained release of L-Arg from the gels indicates the maintenance of a therapeutically relevant concentration over an extended period, suggesting that L-Arg could promote angiogenesis both within the hydrogel matrix and in the surrounding injured tissues. Moreover, L-Arg would be expected to promote muscle regeneration through the regulation of signaling pathways, such as nuclear factor-κB.

### 2.5. In Vitro Cell Compatibility and Proliferation of the Hydrogels

**Figure 5:**
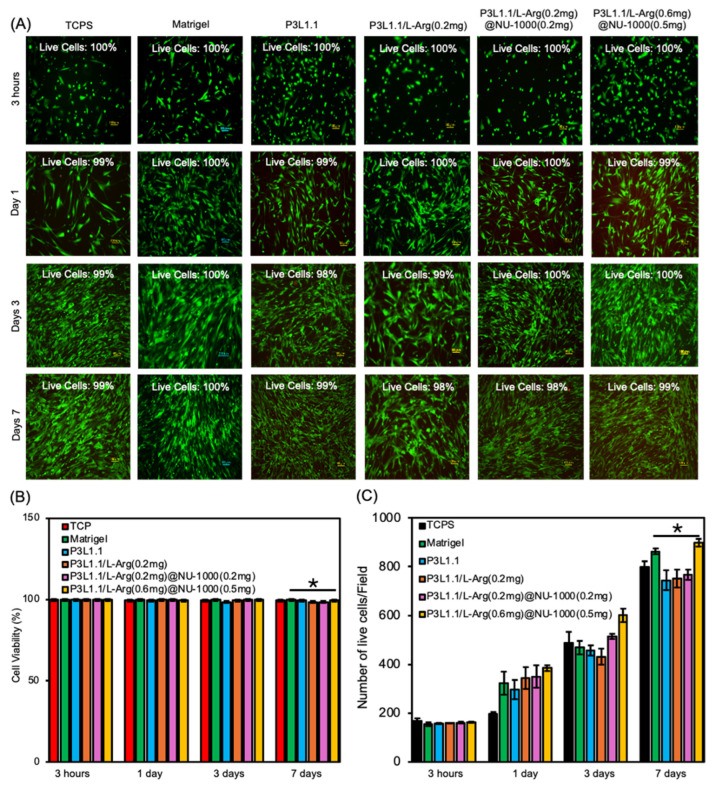
(**A**) Representative fluorescence microscopy images of the HsKMSCs cultured for 1, 3, and 7 days in the presence of the PLGA-PEG-PLGA/LAPONITE^®^/L-Arg@NU-1000 gels, the control PLGA-PEG-PLGA/LAPONITE^®^ gel, and Matrigel. Scale bars: 100 μm. (**B**) Percent quantification from the proliferation of HsKMSC culture. (**C**) Proliferation of the cultured HsKMSCs in the presence of the PLGA-PEG-PLGA/LAPONITE^®^/L-Arg@NU-1000 gels, the control PLGA-PEG-PLGA/LAPONITE^®^ gel, and Matrigel. The *p*-values are indicated as follows: * *p* < 0.05.

Based on the slow-release property of L-Arg, we next investigated the cytocompatibility of the hydrogels using HsKMSCs. For this experiment, the HsKMSCs were cultured in the presence of the gels for 3 h and 1, 3, and 7 days; then, cytocompatibility was assessed using a live/dead cell assay (Figure 5A). From this experiment onward, Matrigel was used as a positive control. The quantitative analysis showed that the cell viability in the injectable hydrogel system P3L1.1/L-Arg(0.6 mg)@NU-1000(0.5 mg) was approximately 99%, closely comparable to that observed in the Matrigel control 100% (Figure 5B). The HsKMSCs exhibited almost 100% viability in the presence of the PLGA-PEG-PLGA/LAPONITE^®^/L-Arg@NU-1000 gels, regardless of the amount of combined L-Arg@NU-1000, indicating that NU-1000 and L-Arg at the specific concentrations used in this study are compatible with HsKMSCs. As expected from these results, the HsKMSCs cultured with the PLGA-PEG-PLGA/LAPONITE^®^/L-Arg@NU-1000 gels exhibited a well-spread morphology. We then examined the proliferation of the HsKMSCs cultured with these gels for 7 days. After 1 and 7 days of culture, the proliferation of the HsKMSCs per field was 324 and 863, respectively, for the Matrigel group, and 385 and 899, respectively, for the P3L1.1/L-Arg (0.6 mg)@NU-1000 (0.5 mg) group. After culturing with the P3L1.1/L-Arg (0.6 mg)@NU-1000 (0.5 mg) gels for 3 days, the HsKMSCs exhibited a higher proliferation ratio compared with the cells cultured with Matrigel, and the proliferation ratio after 7 days was almost the same as that of the cells cultured with Matrigel (Figure 5B). Before performing these experiments, we were concerned about the potential negative effects of NU-1000 on cell proliferation, but we found that the PLGA-PEG-PLGA/LAPONITE^®^ gels containing an appropriate concentration of combined NU-1000 served as an excellent scaffold for promoting cell proliferation, although the mechanism remains unclear.

**Figure 6:**
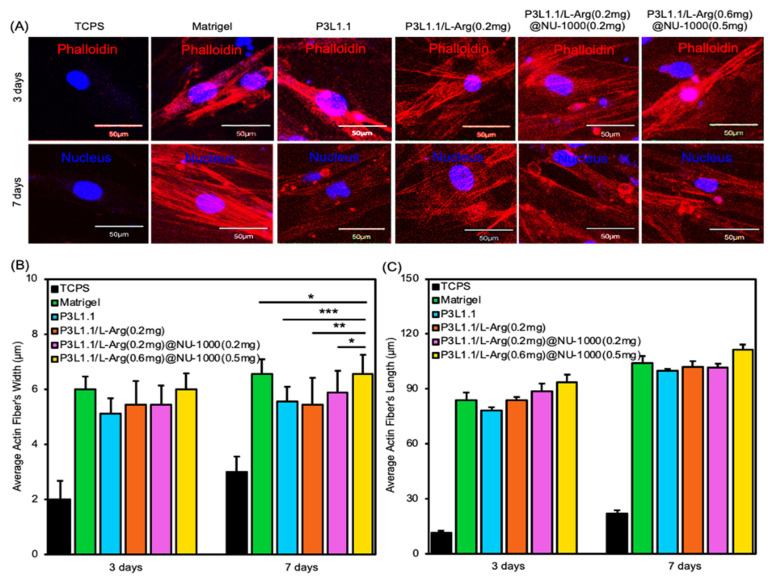
(**A**) Representative fluorescence microscopy images of HsKMSCs stained with phalloidin and DAPI, cultured for 1, 3, and 7 days in the presence of the PLGA-PEG-PLGA/LAPONITE^®^/L-Arg@NU-1000 gels, the control PLGA-PEG-PLGA/LAPONITE^®^ gel, and Matrigel. Scale bars: 50 μm. (**B**) Average width of myotubes detected in the cultured HsKMSCs in the presence of the PLGA-PEG-PLGA/LAPONITE^®^/L-Arg@NU-1000 gels, the control PLGA-PEG-PLGA/LAPONITE^®^ gel, and Matrigel. (**C**) Average length of myotubes detected in the cultured HsKMSCs in the presence of the PLGA-PEG-PLGA/LAPONITE^®^/L-Arg@NU-1000 gels, the control PLGA-PEG-PLGA/LAPONITE^®^ gel, and Matrigel. The *p*-values are indicated as follows: * *p* < 0.05; ** *p* < 0.01; *** *p* < 0.001.

The degree of maturation of actin fibers is an important indicator of the status of HsKMSCs. Therefore, the state of actin fibers in cells cultured in the presence of the hydrogels was then evaluated using phalloidin staining of actin fibers in the cells (Figure 6A). The width and length of each actin fiber formed in the HsKMSCs were determined based on the corresponding images (Figure 6B). Notably, the average width and length of the actin fibers formed in the HsKMSCs cultured in the presence of the P3L1.1/L-Arg (0.6 mg)@NU-1000 (0.5 mg) gels were significantly greater than those of the actin fibers in the HsKMSCs cultured in the presence of the P3L1.1 gels. When compared within the PLGA-PEG-PLGA/LAPONITE^®^/L-Arg@NU-1000 gels, the HsKMSCs cultured with the P3L1.1/L-Arg (0.6 mg)@NU-1000 (0.5 mg) gels containing higher concentrations of L-Arg and NU-1000 exhibited thicker and longer actin fibers. These results are consistent with the results of the cell proliferation assays and suggest that the combination of L-Arg@NU-1000 improves the scaffolding properties of the original PLGA-PEG-PLGA/LAPONITE^®^ gels, as the maturation of actin fibers in HsKMSCs is strongly associated with cell proliferation [66,67,68,69]. Importantly, the 7-day average width and length of the actin fibers formed in the HsKMSCs cultured in the presence of the P3L1.1/L-Arg (0.6 mg)@NU-1000 (0.5 mg) gels, i.e., width of 7 μm and length of 101 μm, were similar to the Matrigel control width of 7 μm and length of 104 μm. These results using the HsKMSCs suggest that our developed gels exhibit good tissue regeneration effects. L-Arginine modulates phalloidin staining in vitro by promoting actin polymerization through post-translational arginylation of β-actin. As a substrate for arginyl-transferase, L-arginine facilitates arginylation, enhancing filamentous actin (F-actin) formation. Mechanically, L-arginine enhances actin filament production in vitro by increasing nitric oxide (NO), which activates the NF-κB signaling pathway to promote actin polymerization [70,71,72].

### 2.6. Evaluation of Skeletal Muscle Tissue Regeneration Capability of the Hydrogels by Immunostaining

**Figure 7:**
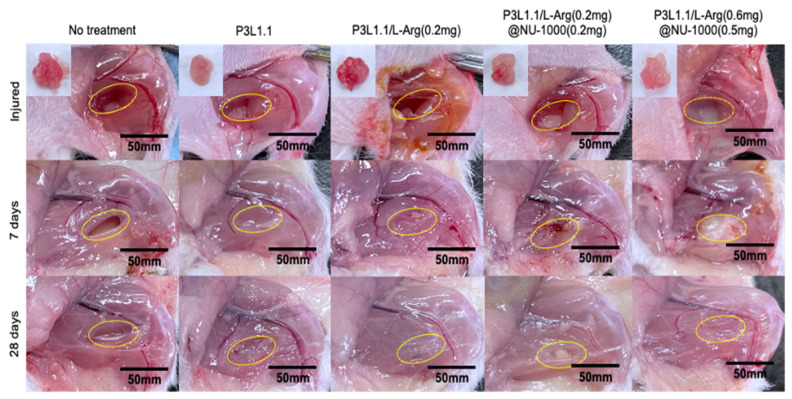
Representative images of injured and reconstructed skeletal muscle tissues after treatments with the PLGA-PEG-PLGA/LAPONITE^®^/L-Arg@NU-1000 gels and the control PLGA-PEG-PLGA/LAPONITE^®^ gels for 7 and 28 days.

**Figure 8:**
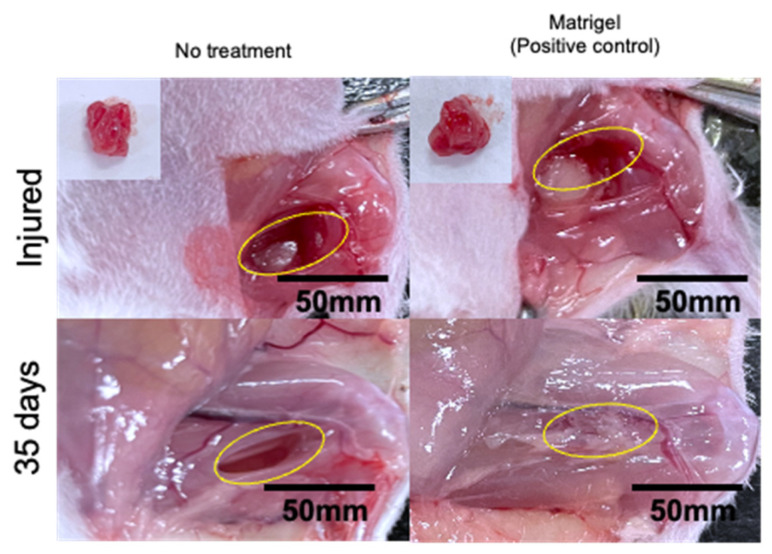
Representative images of injured and reconstructed skeletal muscle tissues after treatments with Matrigel for 35 days.

Based on these results, we proceeded to in vivo skeletal muscle tissue regeneration experiments to compare the regenerative capability of the P3L1.1/L-Arg (0.6 mg)@NU-1000 (0.5 mg) gels with Matrigel. To determine whether the PLGA-PEG-PLGA/LAPONITE^®^/L-Arg@NU-1000 gels promote the regeneration of injured skeletal muscle tissue in vivo by promoting angiogenesis, we first evaluated angiogenesis promotion by the gels using a mouse model of skeletal muscle tissue injury. Additionally, a large area of skeletal muscle tissue was removed from the right hind limbs of the mice using a 4 mm muscle biopsy punch, which resulted in a full-thickness defect muscle mass loss with a width of 2 mm and a depth 4 mm. Approximately, 60 mg of muscle mass was surgically removed from each mouse, and the wound was then closed with sutures, following the injury protocol outlined in Figure S4. Gel precursor solutions were administered via injection into the injured skeletal muscle tissue area. In the absence of the gel treatment, the damaged muscle area did not fill and remained a cavity after even 28 days (Figure 7, area indicated by the dotted line). After 7 days, the biodegradable hydrogels were observed to remain attached to the injured areas in vivo, supporting the degradation process at the injury site. At 28 days, the biodegradable hydrogels completely disappeared from the injured areas in vivo, supporting the regeneration process at the injury site. We demonstrated that the injectable hydrogel samples composed of PLGA-PEG-PLGA/LAPONITE^®^/L-Arg (0.6 mg)@NU-1000 (0.5 mg) were prepared, where PLGA-PEG-PLGA, a biodegradable and thermosensitive triblock copolymer, forms hydrogels in combination with LAPONITE^®^. Upon hydrogel degradation, LAPONITE^®^ facilitates the adsorption of bioactive molecules and promotes tissue regeneration. After 28 days, in the mice treated with the P3L1.1, P3L1.1/L-Arg (0.2 mg), and P3L1.1/L-Arg (0.2 mg)/NU-1000 (0.2 mg) gels, the whitish abnormal tissue appeared reconstructed, with a clear border between the injury and surrounding normal muscle tissue, suggesting lower blood flow in injured tissue than normal muscle tissue. Similar abnormal tissue reconstruction was also observed in the mice treated with Matrigel (Figure 8). After 28 days, the biodegradable hydrogels were degraded in the injured areas in vivo, supporting the degradation process at the injury site. By contrast, the mice treated with the P3L1.1/L-Arg (0.6 mg)/NU-1000 (0.5 mg) gels showed reconstruction of the injured tissue, which appeared reddish in color and had an unclear border with the surrounding normal muscle tissue, suggesting blood flow in the injured tissue was comparable to normal muscle tissue.

**Figure 9:**
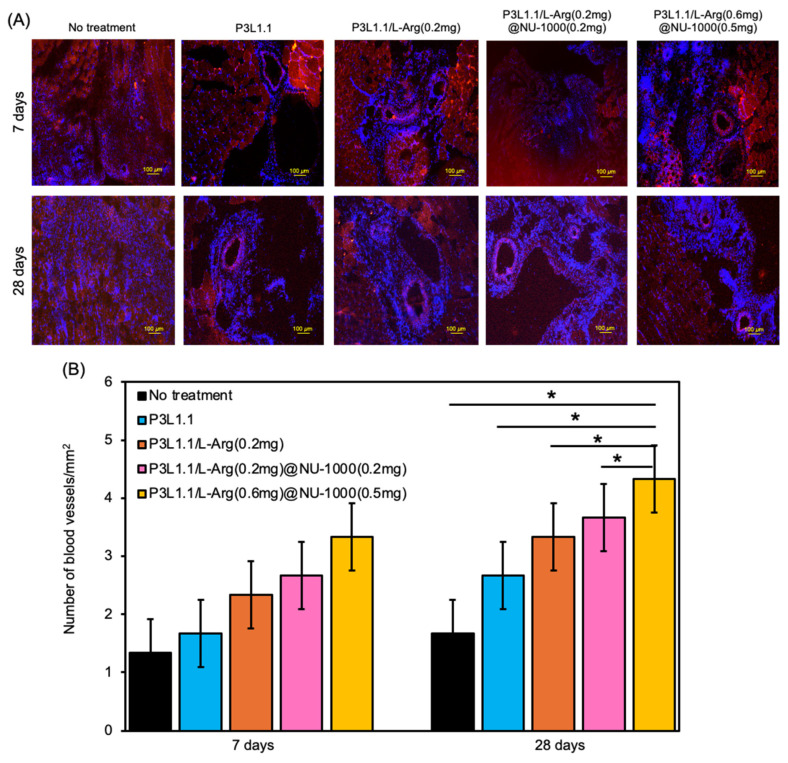
(**A**) Representative fluorescence microscopy images of reconstructed skeletal muscle tissue sections stained with CD31 after 7 and 28 days with treatments by the PLGA-PEG-PLGA/LAPONITE^®^/L-Arg@NU-1000 gels and the control PLGA-PEG-PLGA/LAPONITE^®^ gels. Scale bars: 100 μm. (**B**) Average number of CD31-positive blood vessels in the reconstructed skeletal muscle tissue section after 7 and 28 days with treatments by the PLGA-PEG-PLGA/LAPONITE^®^/L-Arg@NU-1000 gels and the control PLGA-PEG-PLGA/LAPONITE^®^ gels. The *p*-values are indicated as follows: * *p* < 0.05.

**Figure 10:**
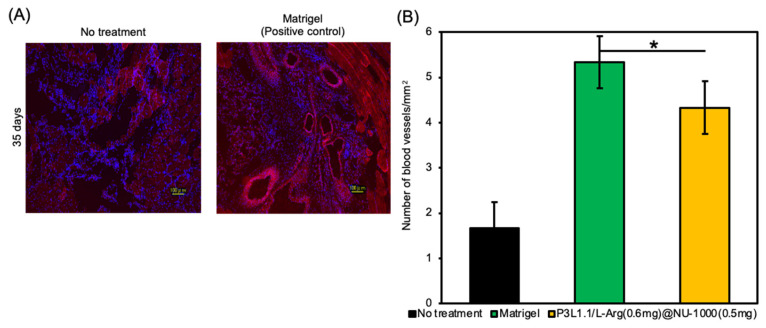
(**A**) Representative fluorescence microscopy images of reconstructed skeletal muscle tissue sections stained with CD31 after 35 days with treatments by Matrigel. Scale bars: 100 μm. (**B**) Average number of CD31-positive blood vessels in the reconstructed skeletal muscle tissue section with treatments by the PLGA-PEG-PLGA/LAPONITE^®^/L-Arg@NU-1000 gels and Matrigel. The *p*-values are indicated as follows: * *p* < 0.05. Significant changes were identified by comparing each with mean 2% ± SD.

To obtain more detailed information regarding angiogenesis, we prepared sections of reconstructed tissue after 7 and 28 days and performed immunofluorescence staining experiments with an anti-CD31 antibody (Figure 9A). As expected from macroscopic external images of the injury sites, only a few CD31-positive new blood vessels were detected in the tissue reconstructed without treatment or the tissue treated with the P3L1.1 gels. The number of new blood vessels was clearly increased by the loading of L-Arg, and when compared within the L-Arg@NU-1000 combined gels, the tissue treated with the P3L1.1/L-Arg (0.6 mg)/NU-1000 (0.5 mg) gels, which contained high concentrations of L-Arg and loaded NU-1000, showed the highest number of new blood vessels (Figure 9B). The Matrigel-treated tissue showed a slightly higher number of new blood vessels than the tissue treated with the P3L1.1/L-Arg (0.6 mg)/NU-1000 (0.5 mg) gels (Figure 10). However, the capability of the P3L1.1/L-Arg (0.6 mg)/NU-1000 (0.5 mg) gels to stably load L-Arg in NU-1000 and deliver it to target injured muscle tissue in the body represents a distinct advantage.

**Figure 11:**
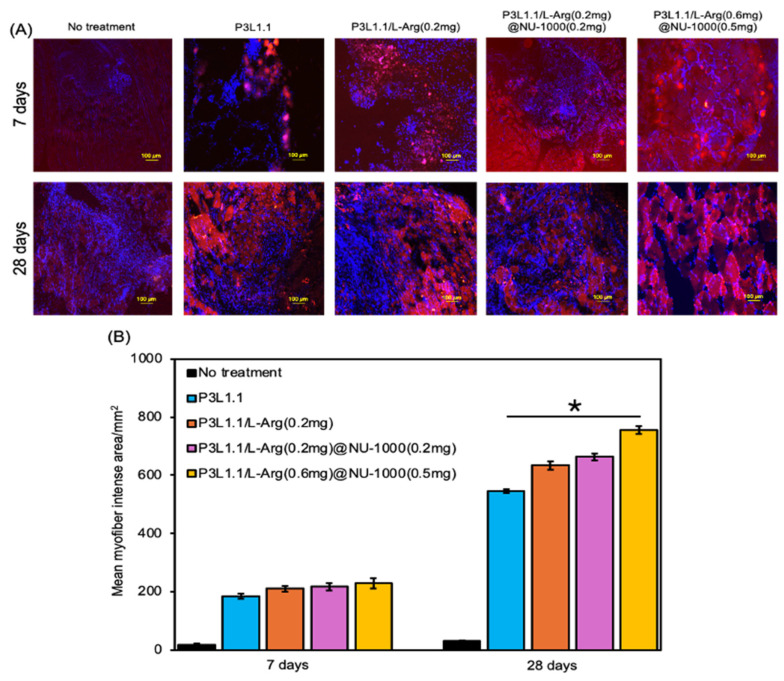
(**A**) Representative fluorescence microscopy images of reconstructed skeletal muscle tissue sections stained with MYH3 after 7 and 28 days with treatments by the PLGA-PEG-PLGA/LAPONITE^®^/L-Arg@NU-1000 gels and the control PLGA-PEG-PLGA/LAPONITE^®^ gels. Scale bars: 100 μm. (**B**) Average number of MYH3-positive myofibers in the reconstructed skeletal muscle tissue section after 7 and 28 days with treatments by the PLGA-PEG-PLGA/LAPONITE^®^/L-Arg@NU-1000 gels and the control PLGA-PEG-PLGA/LAPONITE^®^ gels. The *p*-values are indicated as follows: * *p* < 0.05.

**Figure 12:**
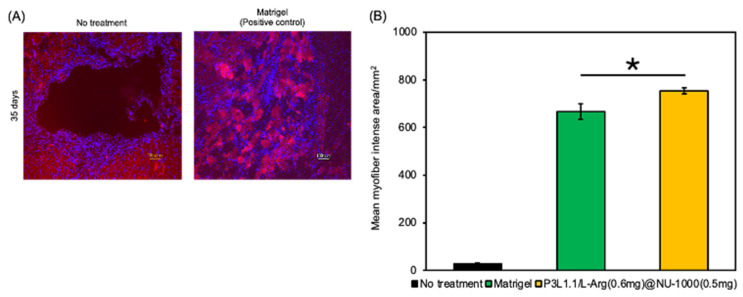
(**A**) Representative fluorescence microscopy images of reconstructed skeletal muscle tissue sections stained with MYH3 after 35 days with treatments by Matrigel. Scale bars: 100 μm. (**B**) Average number of CD31-positive myofibers in the reconstructed skeletal muscle tissue section with treatments by the PLGA-PEG-PLGA/LAPONITE^®^/L-Arg@NU-1000 gels and Matrigel. The *p*-values are indicated as follows: * *p* < 0.05.

We then examined the formation of myofibers and the skeletal muscle regeneration efficiency of the gels. Sections of reconstructed tissue were prepared at 7 and 28 days after treatment and examined using immunofluorescence staining of myosin heavy chain 3 (MYH3), which regulates muscle growth and energy metabolism and plays an important role in muscle development (Figure 11A). As expected from the macroscopic external images of injury sites, almost no MYH3-positive myofibers were detected in the tissue reconstructed without treatment. By contrast, MYH3-positive myofibers were detected in the tissues reconstructed by treatment with various gels. The number of MYH3-positive myofibers was clearly increased by the loading of L-Arg, and when compared within the L-Arg@NU-1000 combined gels, the tissues treated with the P3L1.1/L-Arg (0.6 mg)/NU-1000 (0.5 mg) gels, which had high concentrations of L-Arg and loaded NU-1000, showed the greatest number of new myofibers (Figure 11B). These results agree well with the results of the analyses of in vivo angiogenesis and suggest that L-Arg also plays a role in promoting myofiber formation. In comparison with Matrigel, the P3L1.1/L-Arg (0.6 mg)/NU-1000 (0.5 mg) gels more strongly promoted myofiber neogenesis (Figure 12A,B).

### 2.7. Evaluation of Skeletal Muscle Tissue Regeneration Capability of the Hydrogels by H&E and Masson’s Trichrome Staining

**Figure 13:**
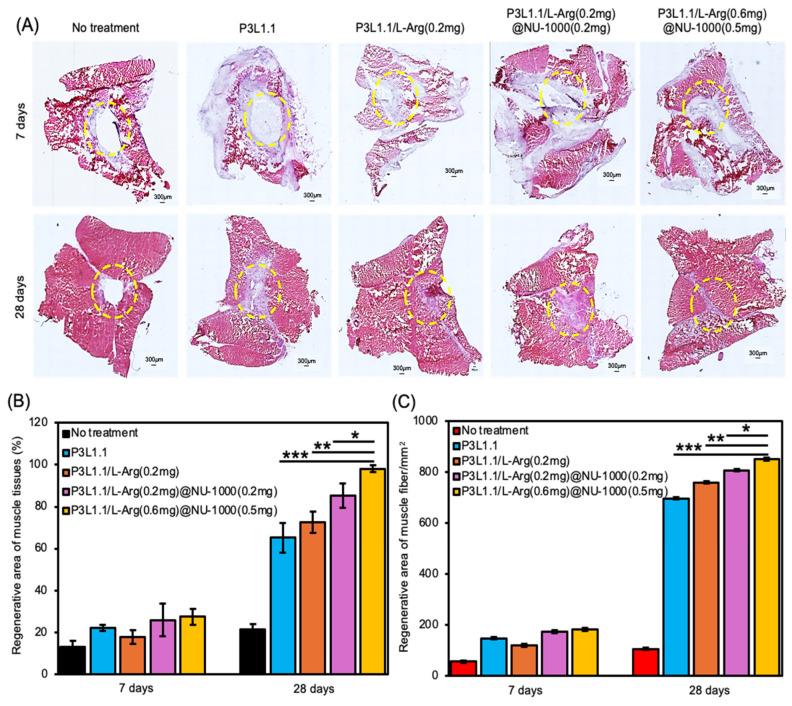
(**A**) Representative microscopy images of reconstructed skeletal muscle tissue sections stained with H&E after 7 and 28 days with treatments by the PLGA-PEG-PLGA/LAPONITE^®^/L-Arg@NU-1000 gels and the control PLGA-PEG-PLGA/LAPONITE^®^ gels. Scale bars: 300 μm. (**B**) Reconstructed area (%) of skeletal muscle tissue sections after 7 and 28 days with treatments by the PLGA-PEG-PLGA/LAPONITE^®^/L-Arg@NU-1000 gels and the control PLGA-PEG-PLGA/LAPONITE^®^ gels. (**C**) Area of the reconstructed skeletal muscle in the whole reconstructed tissues after 7 and 28 days with treatments by the PLGA-PEG-PLGA/LAPONITE^®^/L-Arg@NU-1000 gels and the control PLGA-PEG-PLGA/LAPONITE^®^ gels. The *p*-values are indicated as follows: * *p* < 0.05; ** *p* < 0.01; *** *p* < 0.001.

**Figure 14:**
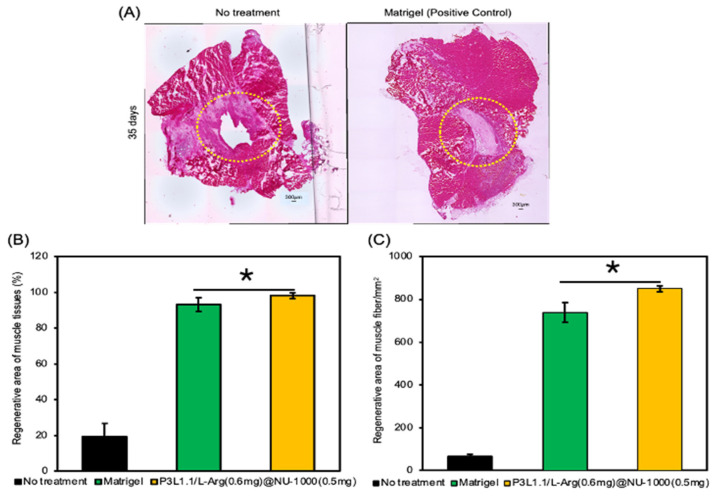
(**A**) Representative microscopy images of reconstructed skeletal muscle tissue sections stained with H&E after 35 days with treatment by Matrigel. Scale bars: 300 μm. (**B**) Reconstructed area (%) in the skeletal muscle tissue section after 35 days with treatment by Matrigel. (**C**) Area of the reconstructed skeletal muscle in whole reconstructed tissues with treatments by the PLGA-PEG-PLGA/LAPONITE^®^/L-Arg@NU-1000 gel and Matrigel. The *p*-values are indicated as follows: * *p* < 0.05.

Next, we evaluated the skeletal muscle regeneration efficiency of the gels using H&E and Masson’s trichrome staining of sections of reconstructed tissues. As expected from the macroscopic external images of injury sites, the damaged muscle area was not filled and remained a cavity even after 28 days in the absence of treatment or treatment with the P3L1.1 gels (Figure 13A). By contrast, after 28 days, in the mice treated with the P3L1.1/L-Arg (0.2 mg) and P3L1.1/L-Arg (0.2 mg)/NU-1000 (0.2 mg) gels, the injured tissues exhibited a clearly distinguishable border with the surrounding normal muscle tissue. Similar abnormal tissue reconstruction was also observed in the mice treated with Matrigel (Figure 14A). By contrast, the mice treated with the P3L1.1/L-Arg (0.6 mg)/NU-1000 (0.5 mg) gels exhibited significantly lower rates of abnormal tissue reconstruction compared with the mice treated with the other gels, including Matrigel (Figure 13A). Compared with the L-Arg@NU-1000 combined gels, the tissues treated with the P3L1.1/L-Arg (0.6 mg)/NU-1000 (0.5 mg) gels, which had high concentrations of L-Arg and loaded NU-1000, showed the greatest regenerative area occupied by the highest muscle fiber content. The injectable hydrogel samples containing P3L1.1/L-Arg (0.6 mg)@NU-1000 (0.5 mg) demonstrated a 1.5-fold increase in the density of muscle fibers, with counts of 851/mm^2^ compared to 731/mm^2^ in the Matrigel control (Figure 13B,C). Importantly, the ratio of regenerated area to area occupied by the reconstructed muscle fibers in the Matrigel-treated tissue was lower than that of the tissue treated with the P3L1.1/L-Arg (0.6 mg)/NU-1000 (0.5 mg) gels (Figure 14B,C).

**Figure 15:**
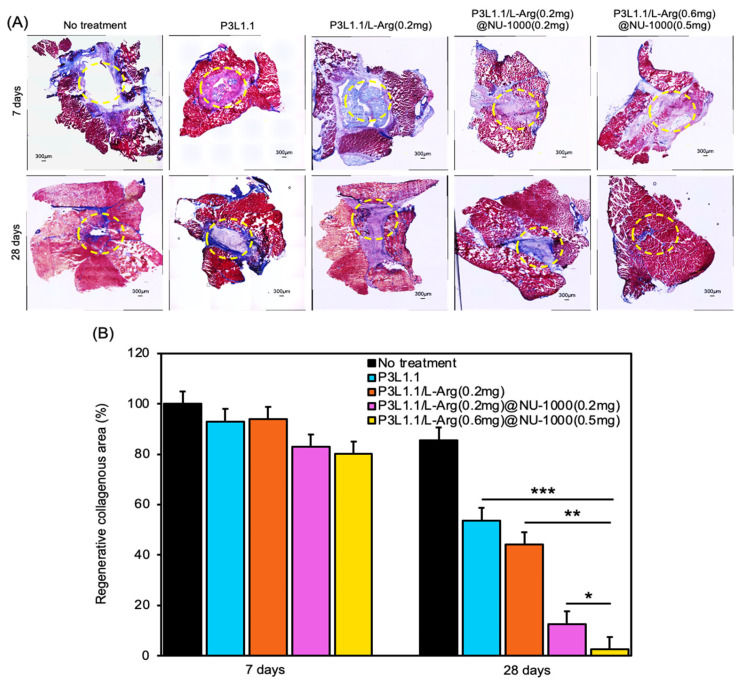
(**A**) Representative microscopy images of reconstructed skeletal muscle tissue sections after Masson trichrome staining, 7 and 28 days after treatments by the PLGA-PEG-PLGA/LAPONITE^®^/L-Arg@NU-1000 hydrogels and the control PLGA-PEG-PLGA/LAPONITE^®^ gels. Scale bars: 300 μm. (**B**) Area of fibrosis in the reconstructed skeletal muscle tissues on 7 and 28 days after treatments by the PLGA-PEG-PLGA/LAPONITE^®^/L-Arg@NU-1000 gels and the control PLGA-PEG-PLGA/LAPONITE^®^ gels. * *p* < 0.05, ** *p* < 0.01, *** *p* < 0.001.

**Figure 16:**
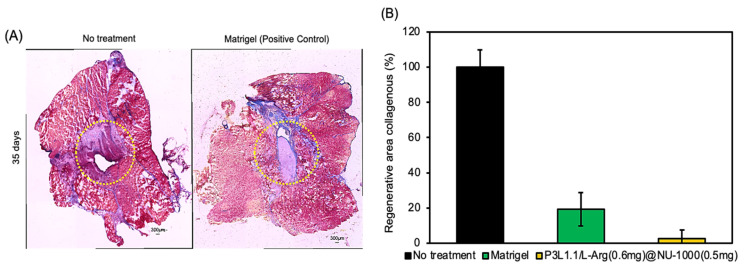
(**A**) Representative microscopy images of reconstructed skeletal muscle tissue sections after Masson trichrome staining, 35 days after treatment by Matrigel. Scale bars: 300 μm. (**B**) Area of fibrosis in the reconstructed skeletal muscle tissues after treatments by the PLGA-PEG-PLGA/LAPONITE^®^/L-Arg@NU-1000 hydrogels and Matrigel.

Masson’s trichrome staining can provide more specific insights into abnormal fibrosis or collagen deposition in muscle tissue during the regeneration process. As expected from the results of the H&E staining, fibrosis sites with abnormal collagen deposition were observed at 28 days in the tissue sections treated with the P3L1.1, P3L1.1/L-Arg (0.2 mg), and P3L1.1/L-Arg (0.2 mg)/NU-1000 (0.2 mg) gels (Figure 15A,B). By contrast, the muscle tissue regenerated by treatment with the P3L1.1/L-Arg (0.6 mg)@NU-1000 (0.5 mg) gels contained almost no fibrotic sites. Notably, compared with Matrigel, treatment with the P3L1.1/L-Arg (0.6 mg)@NU-1000 (0.5 mg) gels resulted in a significantly lower fibrosis induction rate (Figure 16); this reduction in collagen likely reflects a more efficient and functional healing process involving less collagen, thereby leading to better outcomes in skeletal muscle regeneration.

The structure of the muscle tissue regenerated by treatment with the P3L1.1/L-Arg (0.6 mg)@NU-1000 (0.5 mg) gels was similar to that of normal muscle tissue (Figure S5). The results of the analyses of the tissue regeneration efficiency of the P3L1.1/L-Arg (0.6 mg)@NU-1000 (0.5 mg) gels agreed well with the results of CD31 and MHY3 immunofluorescence staining. Moreover, these in vivo results were consistent with the results of in vitro analyses of HsKMSC proliferation and actin fiber formation, indicating that this tissue regeneration effect may be due to the angiogenesis-promoting effect of sustained release of L-Arg, the action of L-Arg on host muscle cells, and the scaffolding effect of NU-1000 for cell growth. Considered collectively, the data of this study suggest that the P3L1.1/L-Arg (0.6 mg)@NU-1000 (0.5 mg) gels provide superior promotion of skeletal muscle tissue regeneration than Matrigel.

## 3. Conclusions

In this study, we developed a fully synthetic injectable hydrogel scaffold by combining a biodegradable PLGA-PEG-PLGA copolymer, LAPONITE^®^ clay nanoparticles, and an L-arginine-encapsulating MOF (NU-1000) at the nano level. The precursor aqueous solution of the developed hybrid gels (PLGA-PEG-PLGA/LAPONITE^®^/L-Arg@NU-1000) undergoes rapid sol–gel transition upon exposure to body temperature, which enables invasive administration of the hydrogels at target sites of tissue injury in the body via simple injection. Long-term, sustained slow release of L-arginine from the resultant hydrogels is possible because NU-1000 serves as a suitable reservoir for L-arginine. The PLGA-PEG-PLGA/LAPONITE^®^/L-Arg@NU-1000 hybrid hydrogels exhibited good cytocompatibility with HsKMSCs and promoted cell growth. Importantly, through in vivo experiments using a mouse model of skeletal muscle injury, we demonstrated that the PLGA-PEG-PLGA/LAPONITE^®^/L-Arg@NU-1000 hydrogels more efficiently promote tissue regeneration than Matrigel. Specifically, we judged the higher tissue regeneration efficacy of our gels by histological analysis, including MYH3 immunofluorescent staining, H&E staining, and Masson’s trichrome staining. Taken together, the results of this study suggest that PLGA-PEG-PLGA/LAPONITE^®^/L-Arg@NU-1000 gels have the potential to become a new type of injectable hydrogel scaffold for in vivo tissue engineering that could replace Matrigel. Considering the variety of biologically active substances that can be encapsulated by NU-1000, PLGA-PEG-PLGA/LAPONITE^®^/NU-1000 gels should have a wide range of applications in regenerative medicine for other tissues. Here, we have attempted to show that the PLGA-PEG-PLGA/LAPONITE^®^/L-arginine@NU-1000 injectable hydrogel system can be adapted to other tissue types beyond skeletal muscle. Based on this discussion and the versatile loading capacities of NU-1000, the PLGA-PEG-PLGA/LAPONITE^®^/NU-1000 hydrogel system can be loaded with proteins, peptides, and various bioactive molecules to target specific tissue regeneration needs, including angiogenesis, osteogenesis, and anti-inflammatory responses [48,73].

## 4. Materials and Methods

### 4.1. Materials

Poly(ethylene glycol) (PEG; MW: 3.0 kDa), glycolide (GA), and tin-2-ethyl hexanoate were purchased from Sigma-Aldrich Japan (Tokyo, Japan) and were ≥99% pure. L-Lactide (L-LA) and D-lactide (D-LA) crystalline were purchased from Musashino Chemical Laboratory Ltd. (Tokyo, Japan) and lyophilized to dry. LAPONITE^®^ was kindly supplied by Wilbur-Ellis Co., Ltd. (Tokyo, Japan). L-Arginine and ninhydrin were purchased from Fujifilm Wako Pure Chemical Co., Ltd. (Osaka, Japan). Zirconium chloride anhydrous and sodium hydroxide (pellets) were purchased from Fujifilm Wako Pure Chemical Co, Ltd. (Osaka, Japan). 4,4′,4′′,4′′′-(Pyrene-1,3,6,8-tetrayl) tetra benzoic acid (H_4_TBAPy) and *N*, *N*-dimethylformamide (DMF) were purchased from Sigma-Aldrich Japan (Tokyo, Japan). 4-Biphenylcarboxylic acid was purchased from Tokyo Chemical Industry Co., Ltd (Tokyo, Japan). We also used other materials received for further purification.

### 4.2. Preparation of NU-1000

To form Zr clusters as nodes of NU-1000, ZrCl_4_ (69.2 mg, 0.297 mmol) and 4-biphenylcarboxylic acid (3.16 g, 15.9 mmol), acting as a modulator, were dissolved in DMF (8 mL) with sonication before heating at 80 °C for 1 h using an aluminum heating block. Meanwhile, H_4_TBAPy (50.3 mg, 73.7 µmol) was dissolved in DMF (3 mL) with sonication before the addition of 1M NaOH aqueous solution (36 µL). After mixing the Zr-based and H_4_TBAPy solutions, the resulting mixture was heated at 100 °C for 24 h. After heating, the resulting samples were filtrated using a 0.2 µm membrane filter, washed three times by soaking in DMF for 24 h, and dried at 120 °C under vacuum to give a slight yellow powder named NU-1000.

### 4.3. Preparation of L-Arginine-Loading NU-1000

First, 1 mg NU-1000 was taken into a glass vial, and 1 mL of L-arginine/Milli-Q water solution (1.5 mg/mL) was added to the vial; then, the suspension was stirred (200 rpm) at 37 °C for the appropriate time (1, 2, 3, 4, 5, 6, 7, 8, 9, 10 days). The resulting samples were washed with Milli-Q water three times and dried under vacuum to give a slight yellow powder named L-arginine-loading NU-1000. For standard preparation, L-arginine at different amounts was taken into a microtube; then, 1 mL Milli-Q water was added to dissolve it, and 1 mL of 4% ninhydrin solution was added. After that, this sample was left for 3 h in a dark place and then measured by a UV-vis spectroscopic analyzer. Loading efficiency = (Loaded amount of L-arginine/Total feed amount of L-arginine) × 100%

### 4.4. Preparation and Characterization of PLGA-PEG-PLGA/LAPONITE^®^/L-Arg@NU-1000 Hydrogels

PLGA-PEG-PLGA was synthesized via bulk ring-opening copolymerization of L-LA, D-LA, and GA in the presence of PEG using tin 2-ethylhexanoate as a catalyst. Briefly, under a nitrogen atmosphere, PEG (2250 mg, 0.75 mmol), L-LA (972 mg, 6.75 mmol), GA (783 mg, 6.75 mmol), and tin 2-ethylhexanoate (12 mg, 30 μmol) were mixed in a glass flask and dried under vacuum overnight. The flask was then purged with nitrogen and sealed tightly. The sealed flask was placed in an oil bath at 150 °C for 6 h. The reaction mixture was dissolved in chloroform and dropped into an excess amount of diethyl ether, and the resultant precipitation was then dried under vacuum overnight to give PLGA-PEG-PLGA. The M_n_ and M_w_/M_n_ of the synthesized copolymers were determined by gel permeation chromatography (GPC) (JASCO, HPLC LC-2000Plus; column, TSK gel; detectors, RI and UV; standard, PEG; eluent, 10 mM LiBr in DMSO). The molecular compositions of PEG, LA, and GA in the copolymers were estimated from ^1^H-NMR measurements (JEOL, ECA-500, solvent: CDCl_3_). First, 100 mg of PLGA-PEG-PLGA copolymer was dissolved in 1 mL of acetone and mixed gently at 200 rpm for 5 min. Then, 1 mL of Milli-Q pure water was added drop by drop into the solution. The total mixture was gently stirred overnight at 100 rpm, and acetone was gradually evaporated by aspiration under stirring to prepare an aqueous solution of PLGA-PEG-PLGA (20%). A specific amount of L-arginine-loading NU-1000 was dispersed in 1 mL of PLGA-PEG-PLGA (3%) solution; then, 1 mL of LAPONITE^®^ (1.1%) aqueous solution was added to each PLGA-PEG-PLGA (3%)/L-arginine@NU-1000 dispersion and vigorously stirred to prepare a hybrid injectable hydrogel solution. The nanocomposites with sol. states were incubated at 4 °C for 10 min, and then their temperature-responsive sol-to-gel transition was investigated by rheometer (MCR302, Anton Paar). The rheological instrument parameter methods of frequency were as follows: 1 rad/s; strain: 4 dyn/c; temperature: 10 °C to 45 °C and 37 °C; and temperature increase rate: 1 °C/60 s to identify the potentiality of the injectable hydrogels.

### 4.5. In Vitro Injectability Properties of PLGA-PEG-PLGA/LAPONITE^®^/L-Arg@NU-1000 Hydrogels

PLGA-PEG-PLGA (3%)/LAPONITE^®^ (1.1%)/L-Arg@NU-1000 hydrogels, 1 mL in total, were prepared in 1 mL syringes. After that, 20 mL vials were filled with 37 °C temperature Milli-Q water. Then, a 1 mL syringe containing P3L1.1 and P3L1.1/L-Arg (0.6 mg)@NU-1000 (0.5 mg) of the hydrogels was injected into the 37 °C Milli-Q water. Finally, we visually identified, by naked-eye observation, whether our developing hydrogels were injectable in vitro.

### 4.6. L-Arginine Release Properties of the Hydrogels

For standard preparation, L-arginine in different amounts was added to a microtube; then, 1 mL PBS was added to dissolve it, and 1 mL of 4% ninhydrin solution was added. After that, these samples were left for 3 h in a dark place and measured by a UV-vis spectroscopic analyzer. PLGA-PEG-PLGA (3%)/LAPONITE^®^ (1.1%) L-arginine@NU-1000 hydrogels (330 μL) were prepared in a multiple well plate; then, 1000 μL of PBS (pH 7.4 of Na = 137 mmol and K = 2.7 mmol) was added into the wells and incubated at 37 °C for an appropriate time in days, with repetition for each sample (n = 3). The hydrogel was cylindrical-shaped, measuring 6.5 mm in diameter and 8.5 mm in length. Afterward, 500 μL of the supernatant was collected at predetermined time intervals (0, 1, 3, 5, 7, 9, 11, 13, 15, 17, 19, 21, 23, 25, 27, 29, 31, 33, 35, 37, 38 days), and 500 μL of fresh PBS was added. Then, 500 μL of 4% ninhydrin solution was added to the collected supernatant samples and left in a dark place for 3 h to allow for reactions between L-arginine and ninhydrin. Finally, the resultant samples were treated by 0.22 μm filtration without the L-arginine sample (the supernatant of the P3L1.1 gel was prepared in the same way) as a blank. The samples were measured to calibrate the machine, and then the amount of L-arginine in the supernatant was quantified by UV spectroscopy measurements at room temperature (wavelength: 400 nm).

### 4.7. In Vitro Cytocompatibility of the Hydrogels

PLGA-PEG-PLGA (3%)/LAPONITE^®^ (1.1%)/L-arginine@NU-1000 hydrogels (330 μL) were prepared in the insert of a Transwell plate. After that, human skeletal muscle satellite cells (HsKMSCs) (2.0 × 10^4^) were cultured on the poly-L-lysine-coated 24-well plate in the presence of the hydrogels for 3 h and 1, 3, and 7 days. After that, the HsKMSCs were washed with PBS twice carefully, and a live/dead assay using calcein-AM (0.002 mmol) and PI (0.003 mmol) solutions was performed for one hour under incubation conditions (5% CO_2_ at 37 °C). The number of cells per field and cell viability (%) were quantified from repetitive samples, n = 3, by using the images obtained by fluorescence microscopy (BZ-9000, KEYENCE, Osaka, Japan). Green fluorescence indicated positive live cells, and red fluorescence indicated negative dead cells. The number of cells per field and cell viability was quantified using ImageJ 1.53c software.

### 4.8. Phalloidin Staining of HsKMSCs Cultured with the Hydrogels

PLGA-PEG-PLGA (3%)/LAPONITE^®^ (1.1%)/L-arginine@NU-1000 hydrogels (330 μL) were prepared in the insert of a Transwell plate. After that, HsKMSCs (2.0 × 10^4^) were cultured on the poly-L-lysine-coated 24-well plate in the presence of the hydrogels for 3 and 7 days. The cells were fixed by 4% formaldehyde at room temperature for 10–30 min; then, the cells were treated with 0.1% Triton X-100 solutions 200 μL for 3 min to increase permeability. The cells were washed with PBS twice and stained by Phalloidin and Hoechst dyes. The resulting images were obtained by fluorescence microscopy (BZ-9000, KEYENCE, Osaka, Japan).

### 4.9. Skeletal Muscle Tissue Regeneration Test

All experimental protocols and animal handling procedures were approved by the Institutional Animal Care and Use Committee of Konan University (K-23-05). Mice (ICR Crl/CD1, 4 weeks old, female) were anesthetized with isoflurane 0.2% vapor condition and shaved to remove the right back side leg hair follicles. Additionally, a large area of skeletal muscle tissue was removed from the right hind limb of the mice using a 4 mm muscle biopsy punch, which resulted in a full-thickness defect muscle mass loss with a width of 2 mm and a depth of 4 mm. Approximately 60 mg ± 5 of muscle mass was surgically removed from each mouse. Three hundred microliters of the PLGA-PEG-PLGA (3%)/LAPONITE^®^ (1.1%)/L-arginine@NU-1000 hydrogels was injected into the injured site of muscle tissue using a syringe with a 24G needle. The experimental treatment groups included the following injectable hydrogel formulations: Matrigel, P3L1.1, P3L1.1/L-Arg (0.2 mg), P3L1.1/L-Arg (0.2 mg)@NU-1000 (0.2 mg), and P3L1.1/L-Arg (0.6 mg)@NU-1000 (0.5 mg). After 7 and 28 days, the mice were sacrificed, and the hydrogels and the surrounding tissues were carefully excised. Cryosections of the samples were prepared (CryoStar NX70, Thermo Scientific, Kanagawa, Japan).

### 4.10. CD31 Staining of the Tissue Section of the Reconstructed Muscle Tissues

Mice (ICR Crl/CD1, 4 weeks old, female) were anesthetized with isoflurane and shaved to remove the right back side leg hair follicles. Then, the skin was cut, the skeletal muscle tissue was removed from the thigh by using a 4 mm diameter cylindrical biopsy punch, and the removed skeletal muscle tissue was weighed. Lastly, the surgical skin was sutured for wound closure, and the mice were kept overnight for treatment. Three hundred microliters of the PLGA-PEG-PLGA (3%)/LAPONITE^®^ (1.1%)/L-arginine@NU-1000 hydrogels was injected into the injured site of the muscle tissue using a syringe with a 24G needle. After 7 and 28 days, the mice were sacrificed, and the hydrogels and the surrounding tissues were carefully excised. Cryosections of the samples were prepared (CryoStar NX70, Thermo Scientific, Kanagawa, Japan), which were immunostained with Alexa Fluor^®^567-labeled anti-CD31 antibody and then analyzed by fluorescence microscopy (BZ-9000, KEYENCE, Osaka, Japan). The number of newly formed blood vessels in the reconstructed tissues was quantified using the corresponding images. We performed three different experiments in triplicate, and the data were expressed as mean ± SD.

### 4.11. MYH3 Staining of the Tissue Section of the Reconstructed Muscle Tissues

Mice (ICR Crl/CD1, 4 weeks old, female) were anesthetized with isoflurane and shaved to remove the right back side leg hair follicles. Then, the skin was cut, the skeletal muscle tissue was removed from the thigh by using a 4 mm diameter cylindrical biopsy punch, and the removed skeletal muscle tissue was weighed. Lastly, the surgical skin was sutured for wound closure, and the mice were kept overnight for treatment. Three hundred microliters of the PLGA-PEG-PLGA (3%)/LAPONITE^®^ (1.1%)/L-arginine@NU-1000 hydrogels was injected into the injured site of muscle tissue using a syringe with a 24G needle. After 7 and 28 days, the mice were sacrificed, and the hydrogels and the surrounding tissues were carefully excised. Cryosections of the samples were prepared (CryoStar NX70, Thermo Scientific, Kanagawa, Japan), which were immunostained with Alexa Fluor^®^567-labeled anti-MYH3 antibody and then analyzed by fluorescence microscopy (BZ-9000, KEYENCE, Osaka, Japan). The fluorescence intensity of MYH3 was quantified by ImageJ using the corresponding images. We performed the three different experiments in triplicate, and the data were expressed as mean ± SD.

### 4.12. H&E Staining of the Tissue Section of the Reconstructed Muscle Tissues

Mice (ICR Crl/CD1, 4 weeks old, female) were anesthetized with isoflurane and shaved to remove the right back side leg hair follicles. Then, the skin was cut, the skeletal muscle tissue was removed from the thigh by using a 4 mm diameter cylindrical biopsy punch, and the removed skeletal muscle tissue was weighed. Lastly, the surgical skin was sutured for wound closure, and the mice were kept overnight for treatment. Three hundred microliters of the PLGA-PEG-PLGA (3%)/LAPONITE^®^ (1.1%)/L-arginine@NU-1000 hydrogels was injected into the injured site of muscle tissue using a syringe with a 24G needle. After 7 and 28 days, the mice were sacrificed, and the hydrogels and the surrounding tissues were carefully excised. Cryosections of the samples were prepared (CryoStar NX70, Thermo Scientific, Kanagawa, Japan). After 30 min of drying, the cut tissue samples were washed with portable water for 5 min until clean and then placed into hematoxylin for 10 min. Then, the samples were washed with 40 °C warm water for 1 min and again washed with portable water for 3 min. Then, the samples were stained with eosin for 5 min, followed by soap. The slides were then placed in 70% ethanol for 1 min, followed by 95% ethanol for 1 min, and 100% ethanol for 10 min. Finally, the samples were treated with xylene for 5 min twice. Then, they were analyzed by fluorescence microscopy (BZ-9000, KEYENCE, Osaka, Japan). The area (%) for the reconstructed muscle tissue was quantified by ImageJ using the corresponding images. We performed the three different experiments in triplicate, and the data were expressed as mean ± SD.

### 4.13. Masson Trichrome Staining of Tissue Sections of the Reconstructed Muscle Tissues

Mice (ICR Crl/CD1, 4 weeks old, female) were anesthetized with isoflurane and shaved to remove the right back side leg hair follicles. Then, the skin was cut, the skeletal muscle tissue was removed from the thigh by using a 4 mm diameter cylindrical biopsy punch, and the removed skeletal muscle tissue was weighed. Lastly, the surgical skin was sutured for wound closure, and the mice were kept overnight for treatment. Three hundred microliters of the PLGA-PEG-PLGA (3%)/LAPONITE^®^ (1.1%)/L-arginine@NU-1000 hydrogels was injected into the injured site of muscle tissue using a syringe with a 24G needle. After 7 and 28 days, the mice were sacrificed, and the hydrogels and the surrounding tissues were carefully excised. Cryosections of the samples were prepared (Thermo Scientific, CryoStar NX70). The cut tissue samples were dried with cool air for 30 min, and then Masson trichrome staining was performed. Bouin’s solution was used as a Masson trichrome kit for tissue mordanting (pre-treatment) for 30 min. After the preparation of the cut tissue slides, they were placed in water for 5 min. Then, 30 min of soap was put into the formaldehyde solutions and washed with water. The Bouin’s solution was heated to 60 °C in a water bath. Then, the washed slides were placed in the Bouin’s solution for 1 h, cooled to room temperature for 10 min, and washed with tap water for 5 min. After washing, the slides were placed in 70% ethanol for the first time for 15 min and then washed to discard the 70% ethanol. The second time, new 75% ethanol was added to the glass slides with the tissue samples for 15 min. Then, the samples were washed with elix water once, and the glass slides were placed into hematoxylin for 5 min. After that, the slides were washed with water for 2 min. After passing the falls, the slides were placed into the fixing solution for 2 min and then cleaned once with elix water. Then, linolenic acid treatment was performed for 15 min, followed by aniline blue for 7 min. After treatment, the slides were washed once with water and then placed into an acetic acid container for 4 min. Then, the slides were washed twice with 95% ethanol for 1 min. Finally, the slides were washed with 100% ethanol solution for 10 min and xylene solution for 10 min. Then, the slides were analyzed by fluorescence microscopy (BZ-9000, KEYENCE, Osaka, Japan). The reconstructed collagenous area was quantified by ImageJ using the corresponding images. We performed the three different experiments in triplicate, and the data were expressed as mean ± SD.

## Figures and Tables

**Table 1:** Gelation temperatures, storage moduli, and loss moduli at 37 °C of a series of PLGA-PEG-PLGA/LAPONITE^®^/L-Arg@NU-1000 hybrid injectable hydrogels (n = 3).

Hydrogels	Gelation Temperature (°C)	Storage Moduli at 37 °C (Pa)	Loss Moduli at 37 °C (Pa)
P3L1.1	19 ± 3.6	141 ± 41	31 ± 12
P3L1.1/L-Arg (0.2 mg)	21 ± 1.0	181 ± 82	47 ± 18
P3L1.1/L-Arg (0.2 mg)@NU-1000 (0.2 mg)	15 ± 0.6	124 ± 49	30 ± 13
P3L1.1/L-Arg (0.6 mg)@NU-1000 (0.5 mg)	18 ± 3.5	237 ± 72	116 ± 17

## Data Availability

All the data supporting the findings presented in this manuscript and the Supplementary Materials are available from the corresponding authors (N.K. and T.T.) upon reasonable request.

## Supplementary Materials

The following supporting information can be downloaded at https://www.mdpi.com/article/10.3390/gels11070514/s1: Figure S1: Structures of (A) LAPONITE^®^ and (B) the PLGA-PEG-PLGA copolymer; Figure S2: Synthesis of the PLGA-PEG-PLGA copolymer; Table S1: Characterization of the synthesized PLGA-PEG-PLGA copolymer; Figure S3: IR spectra of NU-1000 and the hydrogels; Figure S4: Representative model digital images demonstrating the in vivo mice injury process; Figure S5: (A) Representative H&E and Masson trichrome staining images of healthy skeletal muscle of mice. (B) Representative H&E and Masson trichrome staining images of injured skeletal muscle at day 0 (just after the injury).
