# Supplementary material for: MOFs—Combining Fully Synthetic Injectable Hydrogel Scaffolds Exhibiting Higher Skeletal Muscle Regenerative Efficiency than Matrigel
Source: Gels. 2025 Jul 2;11(7):514. doi: 10.3390/gels11070514 (PMC12294706; doi:10.3390/gels11070514)
Supplement: Supplementary file 1 [file gels-11-00514-s001.zip › gels-3684316-supplementary.pdf]

Supplementary Materials

MOFs—Combining Fully Synthetic Injectable Hydrogel Scaffolds Exhibiting Higher Skeletal Muscle Regenerative Efficiency than Matrigel

Sobuj Shahidul Islam, Tatsuya Dode, Soma Kawashima, Myu Fukuoka, Takaaki Tsuruoka * and Koji Nagahama *

Department of Nanobiochemistry, Frontiers of Innovative Research on Science and Technology (FIRST), Konan University, 7-1-20 Minatojima-Minamimachi, Chuo-ku, Kobe 650-0047, Japan; d2161501@s.konan-u.ac.jp (S.S.I.); tatsuya.dd@icloud.com (T.D.); m2461007@s.konan-u.ac.jp (S.K.); h8.kinki.meishinkai@gmail.com (M.F.)

* Correspondence: tsuruoka@konan-u.ac.jp (T.T.); nagahama@konan-u.ac.jp (K.N.)

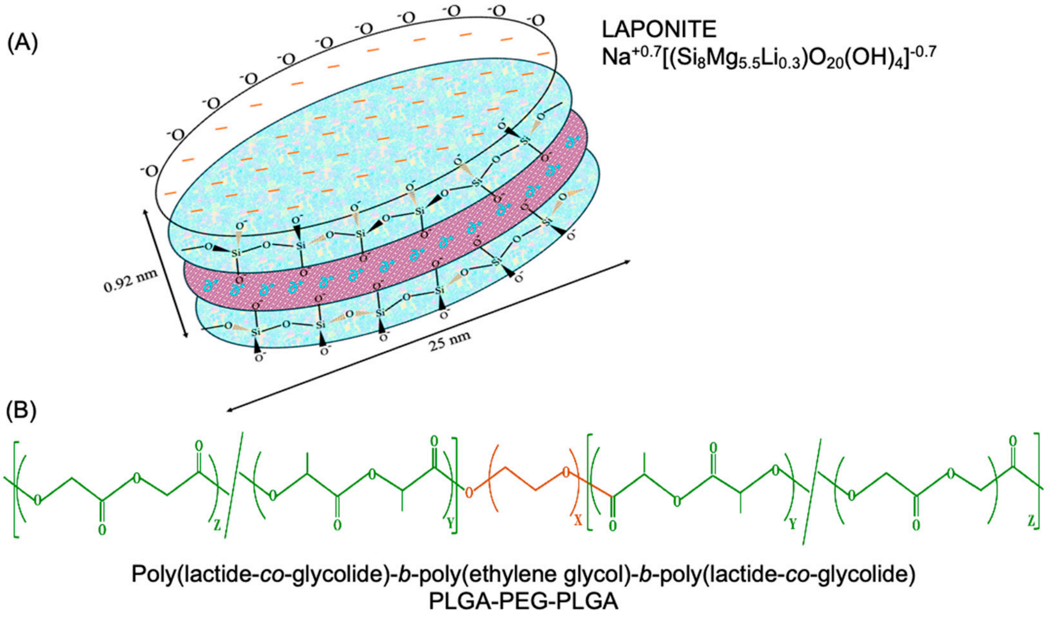

Figure S1. Structures of (A) LAPONITE and (B) PLGA-PEG-PLGA copolymer.

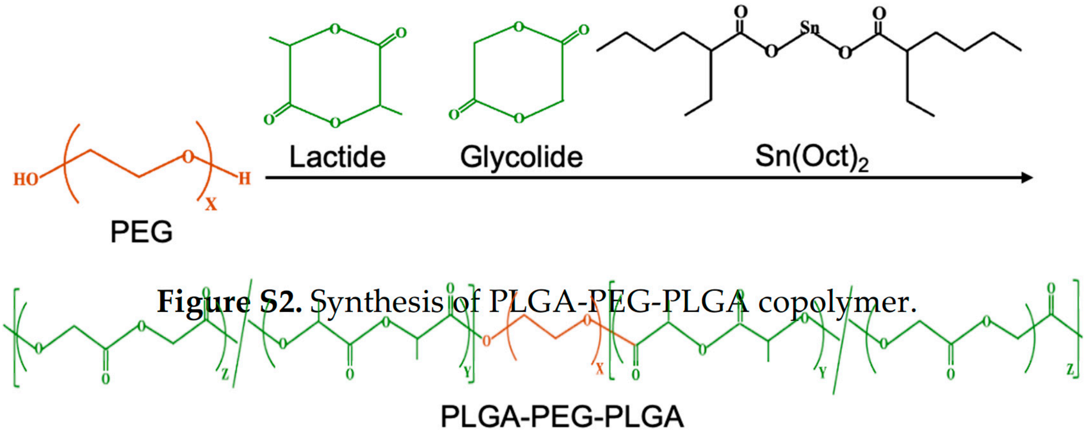

Figure S2. Synthesis of PLGA-PEG-PLGA copolymer.

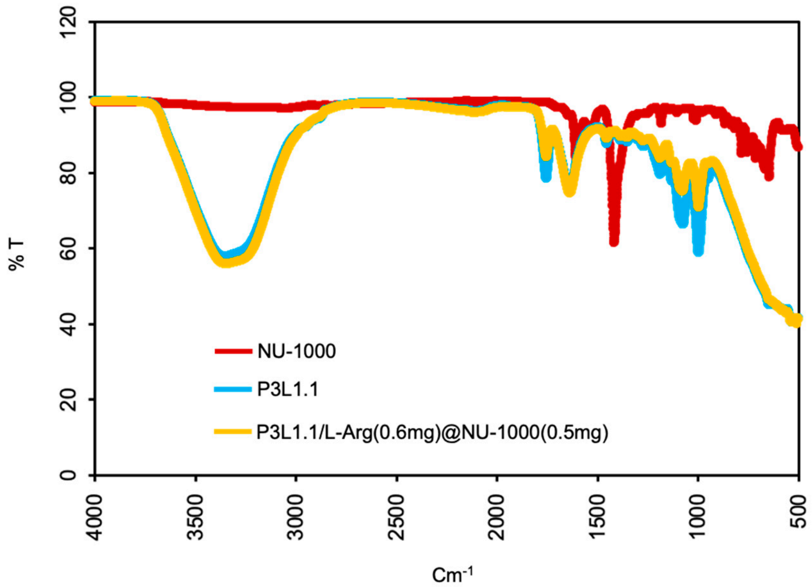

Figure S3. The Infrared Spectroscopic (IR) analysis of injectable hydrogels.

Table S1. Characterization of the synthesized PLGA-PEG-PLGA copolymer.

DP of LA[a]	DP of GA[a]	M_w of PEG	M_n of Copolymer[c]	M_w of Copolymer[c]	M_W / M_n[c]
8	4	3,000	4,960	6,300	1.27

[a] Estimated by ^{1}H-NMR.
[b] Estimated by the following equation: M_w of copolymer = M_w of PEG segment + 2 × (M_w of PLGA segment).
[c] Estimated by GPC (eluent: DMSO, standard: PEG).

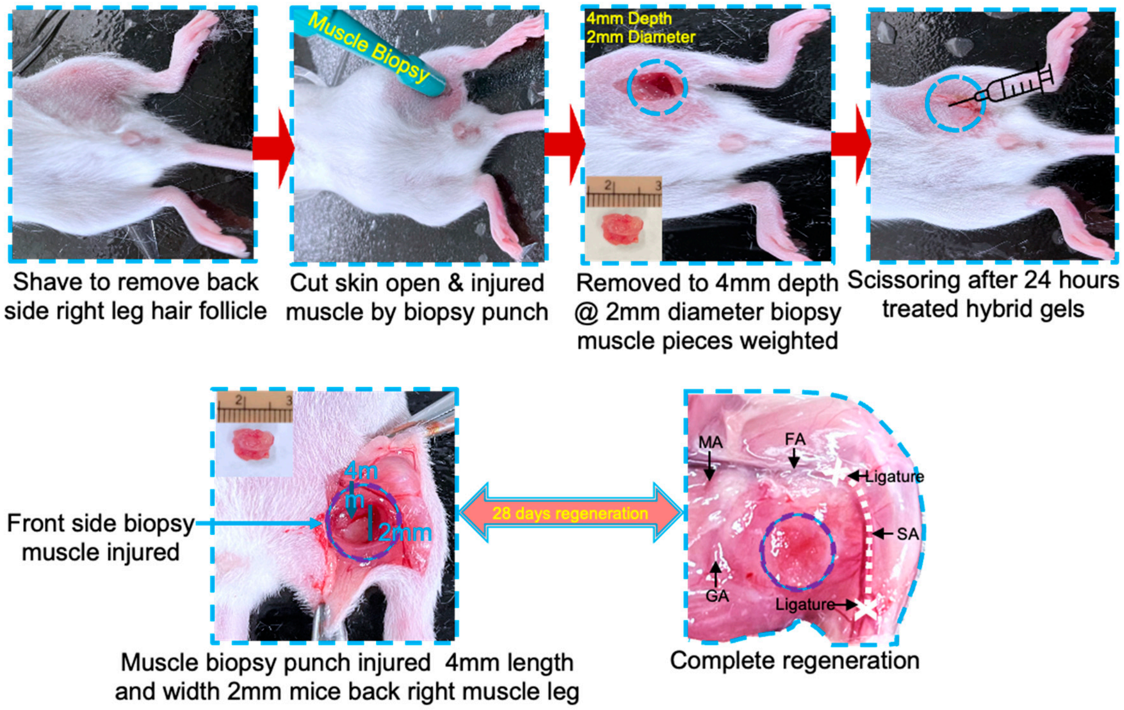

Figure S4. Representative model digital images demonstrated that in vivo mice injured process.

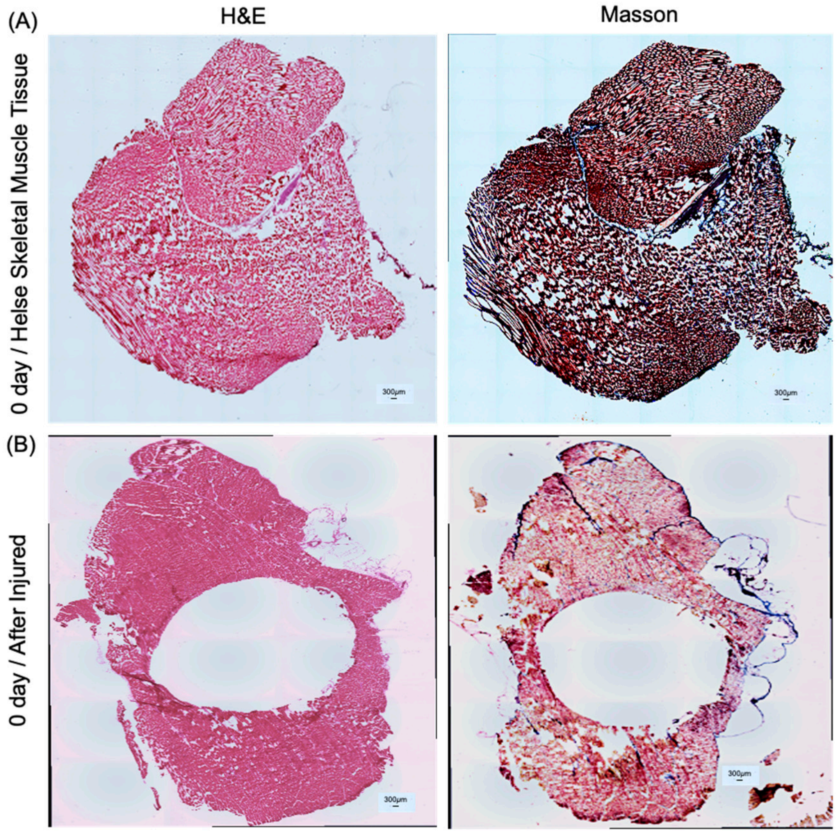

Figure S5. (A) Representative H&E and Masson trichrome staining images of healthy skeletal muscle of mice. (B) Representative H&E and Masson trichrome staining images of injured skeletal muscle at day 0 (just after the injury).
